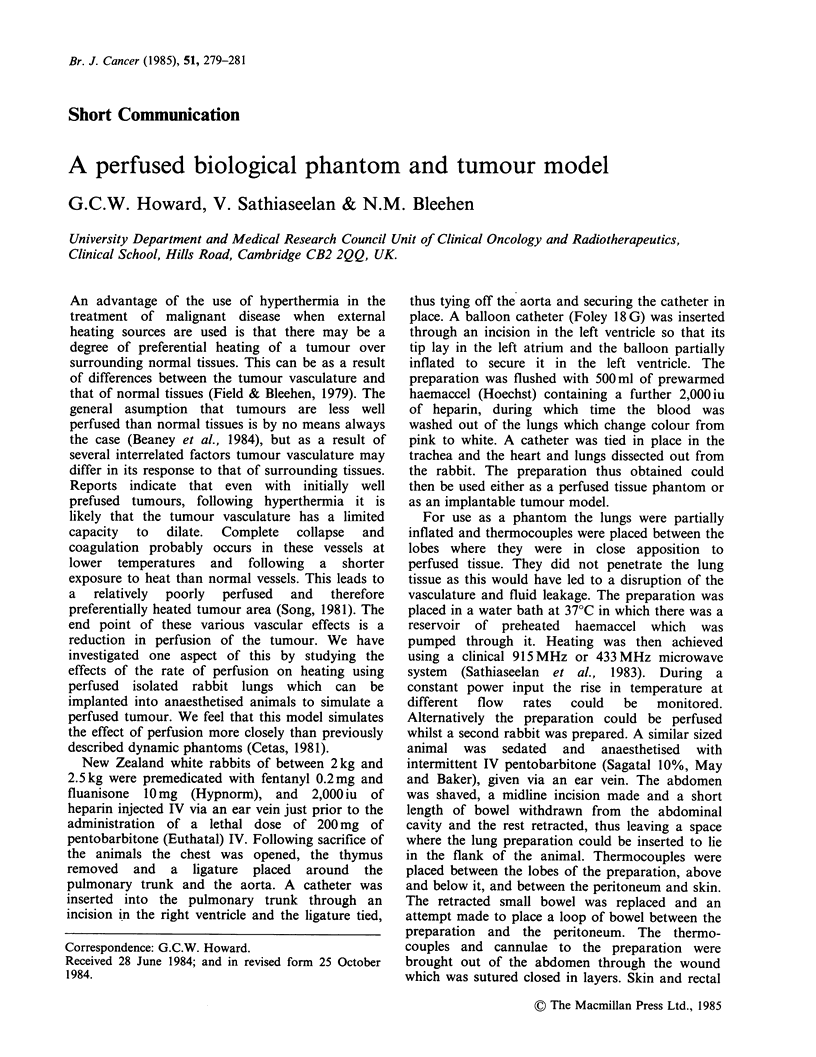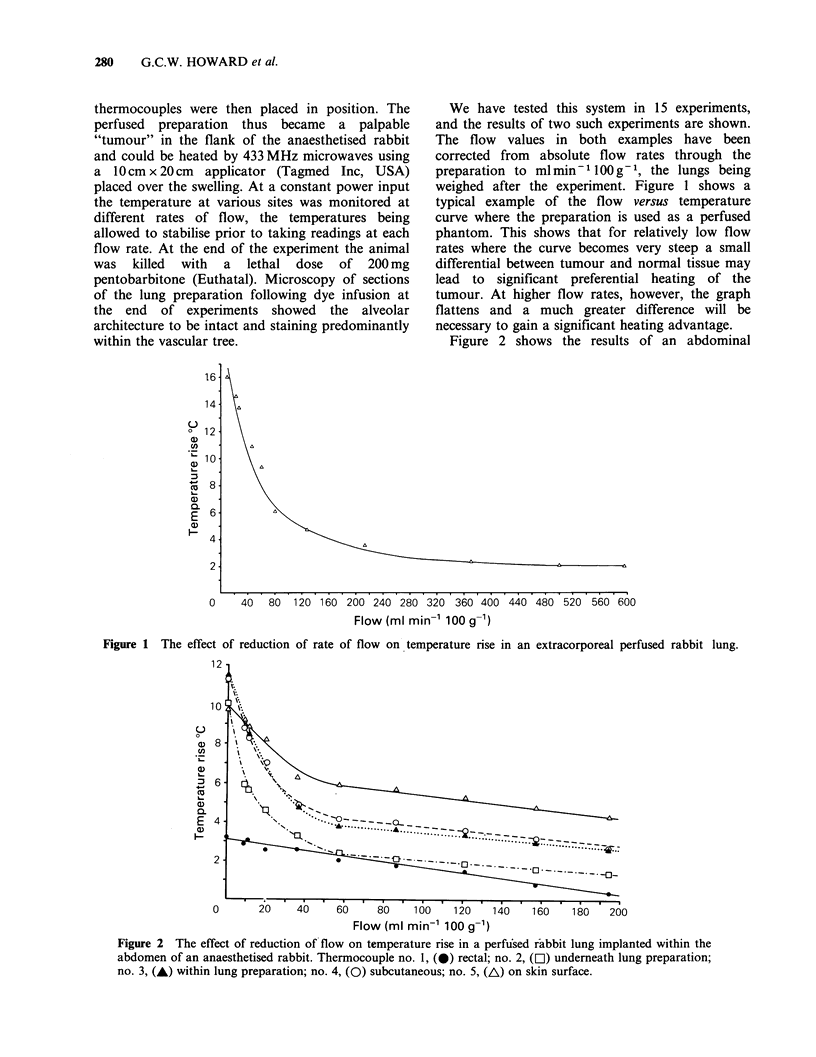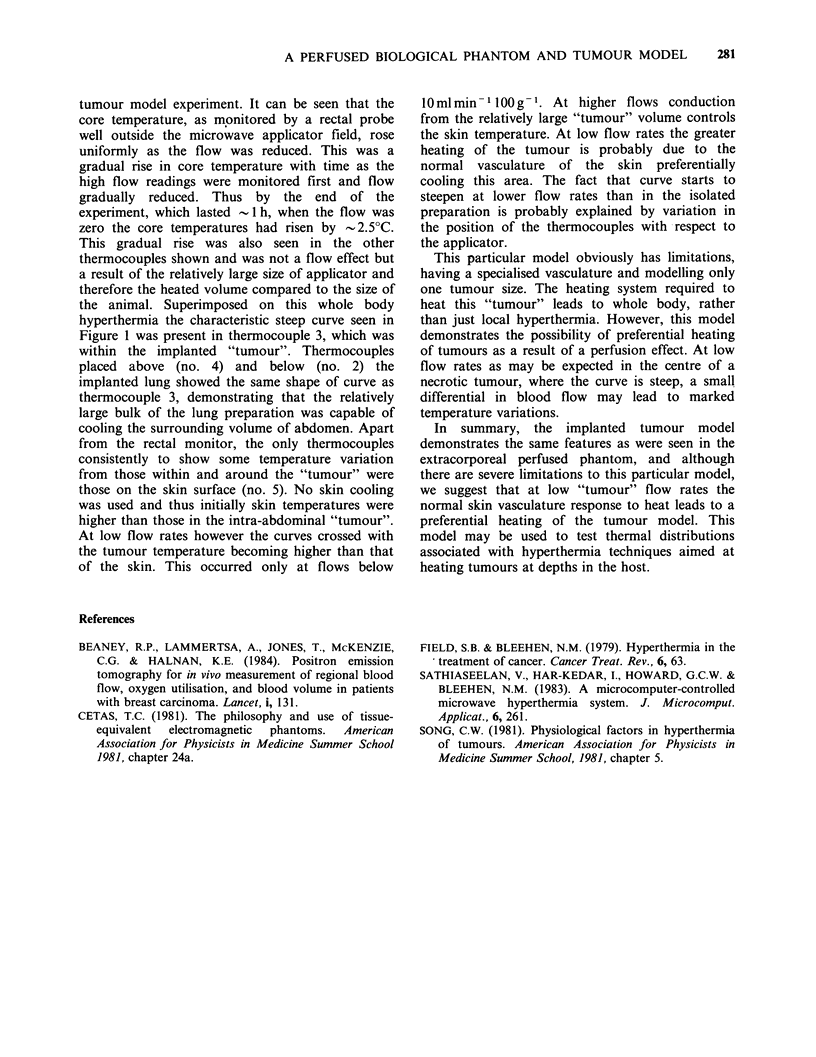# A perfused biological phantom and tumour model.

**DOI:** 10.1038/bjc.1985.40

**Published:** 1985-02

**Authors:** G. C. Howard, V. Sathiaseelan, N. M. Bleehen


*Br. J. Cancer* (1985), **51**, 279–281

### Short Communication

### A perfused biological phantom and tumour model

G.C.W. Howard, V. Sathiaseelan & N.M. Bleehen

*University Department and Medical Research Council Unit of Clinical Oncology and Radiotherapeutics, Clinical School, Hills Road, Cambridge CB2 2QQ, UK.*

An advantage of the use of hyperthermia in the treatment of malignant disease when external heating sources are used is that there may be a degree of preferential heating of a tumour over surrounding normal tissues. This can be as a result of differences between the tumour vasculature and that of normal tissues (Field & Bleehen, 1979). The general asumption that tumours are less well perfused than normal tissues is by no means always the case (Beaney *et al.*, 1984), but as a result of several interrelated factors tumour vasculature may differ in its response to that of surrounding tissues. Reports indicate that even with initially well prefused tumours, following hyperthermia it is likely that the tumour vasculature has a limited capacity to dilate. Complete collapse and coagulation probably occurs in these vessels at lower temperatures and following a shorter exposure to heat than normal vessels. This leads to a relatively poorly perfused and therefore preferentially heated tumour area (Song, 1981). The end point of these various vascular effects is a reduction in perfusion of the tumour. We have investigated one aspect of this by studying the effects of the rate of perfusion on heating using perfused isolated rabbit lungs which can be implanted into anaesthetised animals to simulate a perfused tumour. We feel that this model simulates the effect of perfusion more closely than previously described dynamic phantoms (Cetas, 1981).

New Zealand white rabbits of between 2 kg and 2.5 kg were premedicated with fentanyl 0.2 mg and fluanisone 10 mg (Hypnorm), and 2,000 iu of heparin injected IV via an ear vein just prior to the administration of a lethal dose of 200 mg of pentobarbitone (Euthatal) IV. Following sacrifice of the animals the chest was opened, the thymus removed and a ligature placed around the pulmonary trunk and the aorta. A catheter was inserted into the pulmonary trunk through an incision in the right ventricle and the ligature tied, thus tying off the aorta and securing the catheter in place. A balloon catheter (Foley 18 G) was inserted through an incision in the left ventricle so that its tip lay in the left atrium and the balloon partially inflated to secure it in the left ventricle. The preparation was flushed with 500 ml of prewarmed haemaccel (Hoechst) containing a further 2,000 iu of heparin, during which time the blood was washed out of the lungs which change colour from pink to white. A catheter was tied in place in the trachea and the heart and lungs dissected out from the rabbit. The preparation thus obtained could then be used either as a perfused tissue phantom or as an implantable tumour model.

For use as a phantom the lungs were partially inflated and thermocouples were placed between the lobes where they were in close apposition to perfused tissue. They did not penetrate the lung tissue as this would have led to a disruption of the vasculature and fluid leakage. The preparation was placed in a water bath at 37°C in which there was a reservoir of preheated haemaccel which was pumped through it. Heating was then achieved using a clinical 915 MHz or 433 MHz microwave system (Sathiaseelan *et al.*, 1983). During a constant power input the rise in temperature at different flow rates could be monitored. Alternatively the preparation could be perfused whilst a second rabbit was prepared. A similar sized animal was sedated and anaesthetised with intermittent IV pentobarbitone (Sagatal 10%, May and Baker), given via an ear vein. The abdomen was shaved, a midline incision made and a short length of bowel withdrawn from the abdominal cavity and the rest retracted, thus leaving a space where the lung preparation could be inserted to lie in the flank of the animal. Thermocouples were placed between the lobes of the preparation, above and below it, and between the peritoneum and skin. The retracted small bowel was replaced and an attempt made to place a loop of bowel between the preparation and the peritoneum. The thermocouples and cannulae to the preparation were brought out of the abdomen through the wound which was sutured closed in layers. Skin and rectal

Correspondence: G.C.W. Howard.
Received 28 June 1984; and in revised form 25 October 1984.

© The Macmillan Press Ltd., 1985

thermocouples were then placed in position. The perfused preparation thus became a palpable "tumour" in the flank of the anaesthetised rabbit and could be heated by 433 MHz microwaves using a 10 cm x 20 cm applicator (Tagmed Inc, USA) placed over the swelling. At a constant power input the temperature at various sites was monitored at different rates of flow, the temperatures being allowed to stabilise prior to taking readings at each flow rate. At the end of the experiment the animal was killed with a lethal dose of 200 mg pentobarbitone (Euthatal). Microscopy of sections of the lung preparation following dye infusion at the end of experiments showed the alveolar architecture to be intact and staining predominantly within the vascular tree.

We have tested this system in 15 experiments, and the results of two such experiments are shown. The flow values in both examples have been corrected from absolute flow rates through the preparation to $ml\,min^{-1}\,100\,g^{-1}$, the lungs being weighed after the experiment. Figure 1 shows a typical example of the flow *versus* temperature curve where the preparation is used as a perfused phantom. This shows that for relatively low flow rates where the curve becomes very steep a small differential between tumour and normal tissue may lead to significant preferential heating of the tumour. At higher flow rates, however, the graph flattens and a much greater difference will be necessary to gain a significant heating advantage.

Figure 2 shows the results of an abdominal

**Figure 1** The effect of reduction of rate of flow on temperature rise in an extracorporeal perfused rabbit lung.

**Figure 2** The effect of reduction of flow on temperature rise in a perfused rabbit lung implanted within the abdomen of an anaesthetised rabbit. Thermocouple no. 1, (●) rectal; no. 2, (□) underneath lung preparation; no. 3, (▲) within lung preparation; no. 4, (○) subcutaneous; no. 5, (△) on skin surface.

tumour model experiment. It can be seen that the core temperature, as monitored by a rectal probe well outside the microwave applicator field, rose uniformly as the flow was reduced. This was a gradual rise in core temperature with time as the high flow readings were monitored first and flow gradually reduced. Thus by the end of the experiment, which lasted ~1 h, when the flow was zero the core temperatures had risen by ~2.5°C. This gradual rise was also seen in the other thermocouples shown and was not a flow effect but a result of the relatively large size of applicator and therefore the heated volume compared to the size of the animal. Superimposed on this whole body hyperthermia the characteristic steep curve seen in Figure 1 was present in thermocouple 3, which was within the implanted "tumour". Thermocouples placed above (no. 4) and below (no. 2) the implanted lung showed the same shape of curve as thermocouple 3, demonstrating that the relatively large bulk of the lung preparation was capable of cooling the surrounding volume of abdomen. Apart from the rectal monitor, the only thermocouples consistently to show some temperature variation from those within and around the "tumour" were those on the skin surface (no. 5). No skin cooling was used and thus initially skin temperatures were higher than those in the intra-abdominal "tumour". At low flow rates however the curves crossed with the tumour temperature becoming higher than that of the skin. This occurred only at flows below $10\,ml\,min^{-1}\,100\,g^{-1}$. At higher flows conduction from the relatively large "tumour" volume controls the skin temperature. At low flow rates the greater heating of the tumour is probably due to the normal vasculature of the skin preferentially cooling this area. The fact that curve starts to steepen at lower flow rates than in the isolated preparation is probably explained by variation in the position of the thermocouples with respect to the applicator.

This particular model obviously has limitations, having a specialised vasculature and modelling only one tumour size. The heating system required to heat this "tumour" leads to whole body, rather than just local hyperthermia. However, this model demonstrates the possibility of preferential heating of tumours as a result of a perfusion effect. At low flow rates as may be expected in the centre of a necrotic tumour, where the curve is steep, a small differential in blood flow may lead to marked temperature variations.

In summary, the implanted tumour model demonstrates the same features as were seen in the extracorporeal perfused phantom, and although there are severe limitations to this particular model, we suggest that at low "tumour" flow rates the normal skin vasculature response to heat leads to a preferential heating of the tumour model. This model may be used to test thermal distributions associated with hyperthermia techniques aimed at heating tumours at depths in the host.

### References

BEANEY, R.P., LAMMERTSA, A., JONES, T., McKENZIE, C.G. & HALNAN, K.E. (1984). Positron emission tomography for *in vivo* measurement of regional blood flow, oxygen utilisation, and blood volume in patients with breast carcinoma. *Lancet*, **i**, 131.

CETAS, T.C. (1981). The philosophy and use of tissue-equivalent electromagnetic phantoms. *American Association for Physicists in Medicine Summer School 1981*, chapter 24a.

FIELD, S.B. & BLEEHEN, N.M. (1979). Hyperthermia in the treatment of cancer. *Cancer Treat. Rev.*, **6**, 63.

SATHIASEELAN, V., HAR-KEDAR, I., HOWARD, G.C.W. & BLEEHEN, N.M. (1983). A microcomputer-controlled microwave hyperthermia system. *J. Microcomput. Applicat.*, **6**, 261.

SONG, C.W. (1981). Physiological factors in hyperthermia of tumours. *American Association for Physicists in Medicine Summer School, 1981*, chapter 5.